# Upper Gastrointestinal Hemorrhage: Development of the Severity Score

**DOI:** 10.4021/gr488w

**Published:** 2012-11-20

**Authors:** Rangson Chaikitamnuaychok, Jayanton Patumanond

**Affiliations:** a Department of General Surgery, Kamphaeng Phet Hospital, Kamphaeng Phet, Thailand; b Clinical Epidemiology Unit, Faculty of Medicine, Chiang Mai University, Chiang Mai, Thailand

**Keywords:** Gastrointestinal hemorrhage, Gastrointestinal bleeding, Gastroscopy, Prognostic indicators, Scoring system, Clinical prediction rules

## Abstract

**Background:**

Emergency endoscopy for every patient with upper gastrointestinal hemorrhage is not possible in many medical centers. Simple guidelines to select patients for emergency endoscopy are lacking. The aim of the present report is to develop a simple scoring system to classify upper gastrointestinal hemorrhage (UGIH) severity based on patient clinical profiles at the emergency departments.

**Methods:**

Retrospective data of patients with UGIH in a university affiliated hospital were analyzed. Patients were criterion-classified into 3 severity levels: mild, moderate and severe. Clinical and laboratory information were compared among the 3 groups. Significant parameters were selected as indicators of severity. Coefficients of significant multivariable parameters were transformed into item scores, which added up as individual severity scores. The scores were used to classify patients into 3 urgency levels: non-urgent, urgent and emergent groups. Score-classification and criterion-classification were compared.

**Results:**

Significant parameters in the model were age ≥ 60 years, pulse rate ≥ 100/min, systolic blood pressure < 100 mmHg, hemoglobin < 10 g/dL, blood urea nitrogen ≥ 35 mg/dL, presence of cirrhosis and hepatic failure. The score ranged from 0 to 27, and classifying patients into 3 urgency groups: non-urgent (score < 4, n = 215, 21.2%), urgent (score 4 - 16, n = 677, 66.9%) and emergent (score > 16, n = 121, 11.9%). The score correctly classified 81.4% of the patients into their original (criterion-classified) severity groups. Under-estimation (7.5%) and over-estimation (11.1%) were clinically acceptable.

**Conclusions:**

Our UGIH severity scoring system classified patients into 3 urgency groups: non-urgent, urgent and emergent, with clinically acceptable small number of under- and over-estimations. Its discriminative ability and precision should be validated before adopting into clinical practice.

## Introduction

Upper gastrointestinal hemorrhage (UGIH) is a common challenge encountered in emergency medicine departments. Hospital admissions were approximately 300,000 cases per year in the United States [1]. Case fatalities were also as high as 7-10%, with a yearly expenses of 2.5 billion $US [2].

Endoscopy plays a key role in classifying patients. It is generally suggested that endoscopy should be scheduled within 24 hours after hospital admission [3-6]. The American Society for Gastrointestinal Endoscopy suggested a somewhat earlier timing, within 12 hours [7], but actual early endoscopic examinations were usually scheduled between 2 to 24 hours [8-13]. Almost 80% of UGIH is self-limited [14]. Therefore in most cases endoscopy could be postponed to the following day. This implies that there are only a fraction of patients who actually required an emergency endoscopy. However, this is possible only in hospitals which are 24-hour well-equipped [15]. Clinicians all agreed that endoscopy for patients with mild and moderate UGIH could practically be delayed, and that emergency endoscopy is necessary only for some patients with severe bleeding or those who are in a state of shock. Existing screening procedures for patients with UGIH were mostly based on a scoring system that stratifies patients into those with high or low risk, focusing on ultimate clinical outcomes such as re-bleeding and/or death [16-18]. The purpose of such screening was mainly to help clinicians discharge patients with low risk early and safely, to be treated as out-patients, and to selectively admit patients with high risk to an intensive care unit for close monitoring [16-18].

Our study focused on developing a simple scoring system to predict UGIH severity, by using patient clinical profiles on arrival at the emergency departments, as had been reported earlier [19]. The scores may be used to identify and discriminate UGIH patients with different severity levels without depending entirely on endoscopic examinations and findings.

## Materials and Methods

### Patients and methods

The study was conducted in Kamphaeng Phet Hospital, a university-affiliated tertiary hospital in the northern region of Thailand. We retrieved medical files of patients who presented to the emergency department with upper gastrointestinal bleeding, between 2009 and 2010. The ICD10 keywords for hospital database search were: K920-Hematemesis, K921-Melena and K922-Gastrointestinal hemorrhage unspecified.

### Indicator parameters

The patient profiles of interest were: 1) Demographic profiles: gender and age; 2) Mode of presentation: hematemesis, coffee ground vomiting, hematochezia, melena, and syncope; 3) Hemodynamic profiles: pulse rate and systolic blood pressure; 4) Biochemical profiles: hemoglobin and blood urea nitrogen, and 5) Co-morbidities: presence of cirrhosis, hepatic failure, cardiac failure and renal failure.

### Definitions of UGIH severity: an outcome of interest

We used the following criteria to define UGIH severity: 1) Severe: patients who required surgical interventions to stop bleeding, patients in a state of grade 3 and 4 shock [20], and patients who did not survive; 2) Moderate: patients who required endoscopy to stop bleeding (endotherapy), patients with re-bleeding, patients in a state of grade 1 and 2 shock, and patients who required blood transfusion; 3) Mild: patients with no signs of shock, patients who required endoscopy without hemostasis, and patients who did not required any blood transfusions.

### Data analysis

The patient profiles were compared across the three severity groups by chi-squared tests for linear trends, or two-way ANOVA by rank. Significant indicators for UGIH severity were presented by odds ratios from a multivariable ordinal continuation ratio logistic regression, which is most suitable for ordinal-natured outcomes such as in this study. The significant coefficients were transformed into item scores and added up to a single total score for each patient. The discriminative performances of the scores were displayed graphically. The patients were classified by the scores into 3 urgency groups corresponding to their original severity: non-urgent, urgent and emergent. The score-classification of urgency was compared to the criterion-classification of severity.

The study was approved by The Kamphaeng Phet Hospital Ethical Committee for Clinical Research. No patients inform consents were required for this retrospective data collection. Traceable patient information was omitted in all processes of data analysis and presentations. The authors declared no out-source grants received and no conflicts of interests.

## Results

We retrieved 1,043 medical files corresponding with the above definitions. Among these, 255 were criterion-classified as mild, 664 as moderate, and 124 as severe.

### Significant predictors

By a univariable analysis, patients in the 3 severity groups were similar according to gender (P = 0.871), presence of hematemesis (P = 0.315), hematochezia (P = 0.462), and hepatic failure (P = 0.071), but were different according to age, coffee ground vomiting, melena, syncope, pulse rate, systolic blood pressure, hemoglobin, blood urea nitrogen, presence of cirrhosis, cardiac failure, and renal failure (Table 1).

**Table 1:** Characteristics of Patients With Upper Gastrointestinal Hemorrhage, Criterion-Classified into Three Severity Levels

Patient characteristics	Mild	Moderate	Severe	P-value^*^
	n = 255 mean ± SD	n = 664 mean ± SD	n = 124 mean ± SD	
Demographics				
Male (n, %)	175 (68.6)	427 (64.3)	87 (70.2)	0.871
Age (year)	54.6 ± 18.0	60.4 ± 14.8	58.4 ± 14.2	0.010
Mode of presentation (n, %)				
Hematemesis	117 (45.9)	299 (45.0)	66 (53.2)	0.315
Coffee ground vomiting	67 (26.3)	114 (17.2)	26 (21.0)	0.048
Hematochezia	20 (7.8)	40 (6.0)	8 (6.5)	0.462
Melena	113 (44.3)	421 (63.4)	69 (55.7)	0.001
Syncope	28 (11.0)	144 (21.7)	34 (27.4)	< 0.001
Hemodynamics				
Pulse (/min)	89.8 ± 16.3	91.4 ± 15.7	93.1 ± 17.7	0.022
SBP (mmHg)	128.6 ± 21.6	120.6 ± 20.5	88.5 ± 17.0	< 0.001
Biochemicals				
Hemoglobin (g/dL)	11.4 ± 2.4	7.0 ± 2.1	7.4 ± 2.9	< 0.001
BUN (mg/dL)	23.9 ± 16.5	36.5 ± 21.7	37.6 ± 22.5	< 0.001
Co-morbidities (n, %)				
Cirrhosis	14 (5.5)	106 (16.0)	28 (22.6)	< 0.001
Hepatic failure	0 (0)	6 (0.9)	2 (1.6)	0.071
Cardiac failure	1 (0.4)	6 (0.9)	4 (3.2)	0.024
Renal failure	4 (1.6)	53 (8.0)	12 (9.7)	< 0.001
Clinical outcomes (n, %)				
Re-bleeding	6 (2.4)	42 (6.3)	18 (14.5)	< 0.001
Dead	0 (0)	1 (0.2)	24 (19.4)	< 0.001

*P-value from chi-squared for linear trends, or two-way ANOVA by rank. SD: standard deviation; SBP: systolic blood pressure; BUN: blood urea nitrogen.

Under a multivariable analysis, the remaining patient profiles that significantly increased UGIH severity levels were: age ≥ 60 years (OR = 1.57, 95% CI = 1.13 - 2.18, P = 0.007), pulse rate ≥ 100/min (OR = 1.56, 95% CI = 1.11 - 2.19, P = 0.011), systolic blood pressure < 100 mmHg (OR = 97.49, 95% CI = 54.86 - 173.25, P < 0.001), hemoglobin < 10 g/dL (OR = 15.00, 95% CI = 10.48 - 21.46, P < 0.001), blood urea nitrogen ≥ 35 mg/dL (OR = 2.22, 95% CI = 1.57 - 3.14, P < 0.001), presence of cirrhosis (OR = 2.55, 95% CI = 1.58 - 4.14, P < 0.001) and presence of hepatic failure (OR = 8.12, 95% CI = 1.66 - 39.67, P = 0.010). The first two strongest predictors were systolic blood pressure < 100 mmHg (OR = 97.49) and hemoglobin < 10 g/dL (OR = 15.00) (Table 2).

**Table 2:** Significant Predictors of Upper Gastrointestinal Hemorrhage Severity and Assigned Item Score

Predictors	Category	OR	95% CI	P-value	Coefficient^*^	Score
Age (year)	≥ 60	1.57	1.13 - 2.18	0.007	0.45	1
	< 60	1.00	ref			0
Pulse (/min)	≥ 100	1.56	1.11 - 2.19	0.011	0.44	1
	< 100	1.00	ref			0
Systolic pressure (mmHg)	< 100	97.49	54.86 - 173.25	< 0.001	4.58	10.5
	≥ 100	1.00	ref			0
Hemoglobin (g/dL)	< 10	15.00	10.48 - 21.46	< 0.001	2.71	6
	≥ 10	1.00	ref			0
BUN (mg/dL)	≥ 35	2.22	1.57 - 3.14	< 0.001	0.80	2
	< 35	1.00	Ref			0
Cirrhosis	yes	2.55	1.58 - 4.14	< 0.001	0.94	2
	no	1.00	Ref			0
Hepatic failure	yes	8.12	1.66 - 39.67	0.010	2.09	4.5
	no	1.00	ref			0

*Coefficients from multivariable continuation ratio logistic regression. OR: odds ratio; CI: confidence interval; ref: reference category; BUN: blood urea nitrogen.

### The scoring system

The above significant coefficients were divided by the smallest coefficient (0.44) and rounded up or down to the nearest 0.5 integers to serve as item scores. Scores were not available for 30 patients with missing information of key variables. The item scores ranged from 0 to 10.5 and the sum (total) scores may range from 0 to 27 (Table 2).

### Discriminations

**Figure 1:**
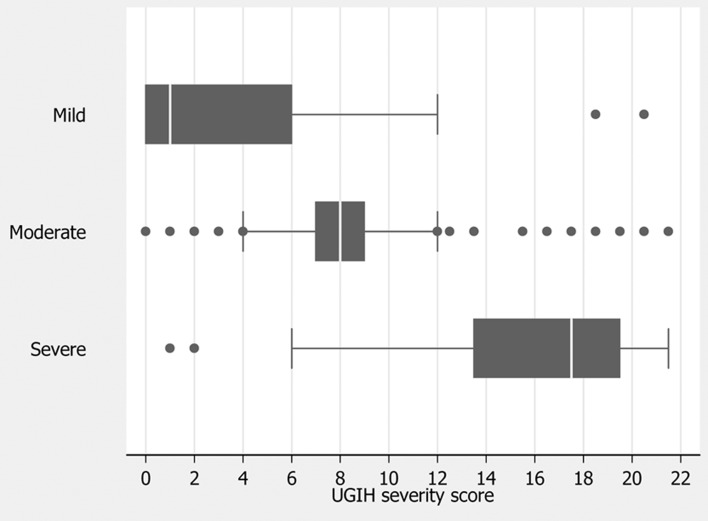
Distribution of UGIH severity scores by criterion-classified severity levels. Vertical lines in box represent medians. Box boundaries represent 25th and 75th percentiles.

**Figure 2:**
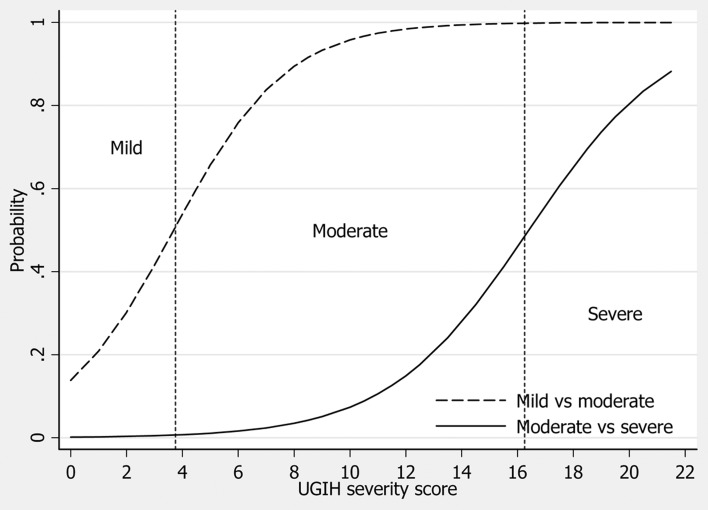
Discrimination of urgency based on UGIH severity scores. Dash line: mild (non-urgent) vs. moderate (urgent). Solid line: moderate (urgent) vs. severe (emergent). Vertical dotted lines represent boundaries (cut-off points) of the scores.

The mean scores for patients in the mild, moderate and severe groups were 3.0 ± 3.5, 8.3 ± 3.3, and 16.1 ± 4.2 respectively (Table 3), and clustered within different distributions (Fig. 1). The derived scores discriminated moderate UGIH from mild UGIH at moderate scores, and discriminated severe UGIH from moderate UGIH at higher scores (Fig. 2).

**Table 3:** Score-Classified Urgency Levels, Criterion-Classified Severity Levels, and Risk Estimation Validity

Score-classified urgency levels		Score range	Criterion-classified severity levels			Risk estimation validity^*^		
			Mild	Moderate	Severe	Over	Correct	Under
			n = 247	n = 650	n = 116	(%)	(%)	(%)
Mean ± SD			3.0 ± 3.5	8.3 ± 3.3	16.1 ± 4.2			
IQR**			0 - 6	7-9	13.5-19.5			
Non-urgent	n = 215	< 4	174	39	2	-	17.2	4.0
Urgent	n = 677	4 - 16	71	571	35	7.0	56.4	3.5
Emergent	n = 121	> 16	2	40	79	4.1	7.8	-
					Total	11.1	81.4	7.5

*Percentage of total patients. SD: standard deviation; IQR: Inter-quartile range.

### Calibrations

For each of the 3 levels of severity, the score-predicted percents were calibrated against the criterion-classified percents. The compared percentages agreed correspondingly, yielding P-values (goodness-of-fit statistics) of 0.992, 0.996 and 0.992 for prediction of mild, moderate and severe UGIH respectively, implying no lack-of-fits.

### Clinical predictions

The score predicted patients who were at least in a moderate group (moderate or severe vs. mild) with high percentage (area under the ROC curve = 0.8813; 95% CI = 0.8600 - 0.9008, data not shown), and predict patients who were in a severe (severe vs. mild or moderate) group with greater proportion (area under the ROC = 0.9274; 95% CI = 0.9092 - 0.9422, data not shown).

For clinical purposes, patients were score-classified into 3 groups: scores < 4, non-urgent; scores 4 - 16, urgent; and scores > 16, emergent. The scores of < 4 correctly classified 174 out of 247 “mild” patients as “non-urgent”, with 1-level under-estimation in 39 patients and 2-level under-estimation in 2 patients (an overall under-estimation of 4.0%). A score of 4 - 16 correctly classified 571 out of 650 “moderate” patients as “urgent”, with 35 cases (3.5%) under-estimation and 71 cases (7.0%) over-estimation. A score of > 16 classified 79 out of 116 “severe” patients as “emergent”, with 40 patients 1-level over-estimation, and 2 patients 2-level over-estimation (an overall over-estimation of 4.1%) (Table 3).

## Discussion

The development of scoring systems for screening patients presenting with UGIH may be classified into 2 groups. The first group used information on clinical and laboratory parameters without endoscopic findings. Examples are the Blatchford Score [21], the Bleed Risk Classification [22, 23], and the Clinical Rockall Score. The second group used endoscopic examinations in addition to clinical and laboratory parameters. Examples are the Complete Rockall Score, the Baylor-College Score [24] and the Cedars-Sinai Score [25]. Advantages or disadvantages of the two scoring systems depend on the patient settings and clinical outcomes to be predicted. The Blatchford Score has high sensitivity in predicting the need for intervention, but its low specificity results in obvious overestimation [26]. The Rockall Score has a good prediction for death, but a poor prediction for re-bleeding, or the need for surgical procedures [27].

The existing scoring systems to classify UGIH patients into severity levels, like the Blatchford Score [21] or the Rockall Score, classified patients into only 2 groups, high risk and low risk. Very narrow ranges of the scores seemed to cause some limitations on clinical practice. In the Blatchford Score, patients scoring 0 were classified as the “low risk” [21, 28], and those scoring 1 or more as the “high risk”. The same rule was also used in the Rockall Score [28, 29]. As there are only a small number of patients in the low risk group, the score classified a large number of patients as “high risk”. For examples, using the Blatchford Score, there would be 92.1% of patients classified as the “high risk”, 81.6% from using the Clinical Rockall Score, and 70.1% by using the Complete Rockall Score [28]. This over classification will results in increasing patient loads and medical resources overuse.

Classifying patients into broader ranges may be more practical for clinicians and surgeons. Our study used 2 cut-off points to classify patients into 3 groups: non-urgent, urgent and emergent. Directive actions are correspondingly suggested as follows: 1) Patients scoring < 4, the “non-urgent” representing “mild” group, had low level of severity. They could be managed conservatively. No blood transfusion may be needed, and elective endoscopy may be appointed in 96 hours to 10 days. These patients may be treated as out-patients. This low risk group correspond to the “low risk” scoring 0 in the Blatchford Score and the Pre-Endoscopic Rockall Score, in which patients were successfully treated as out-patients, the re-bleeding rate was very low and no deaths reported [5, 22]; 2) Patients scoring between 4 and 16, the “urgent” representing the “moderate” group, had a higher severity level. These patients should be admitted to hospital with additional interventions. Resuscitation may be needed. Fluid replacement and/or blood transfusion may be given as indicated. Endoscopy should be appointed within 24 - 96 hours after admission. Patients in this group comprised approximately 60% of all UGIH patients and corresponded to some patients in the high risk group from the Blatchford Score and the Rockall Score [5, 22]; 3) Patients scoring > 16, the “emergent” representing the “severe” group, had the highest severity level. These patients may experience blood pressure drop. Vigorous resuscitation may be needed. They should be admitted to an intensive care unit (ICU) for close monitoring. Endoscopy should be appointed immediately, within 24 hours, or as soon as vital signs are stable. Some patients may require surgical interventions to stop bleeding or for life savings. These patients corresponded to the “high risk” group in both the Blatchford Score and the Rockall Score.

Our algorithm classified 21.2% patients as the mild group, similar percentages to the Blatchford Score and the Rockall Score [22], but the number of patients in the high risk group would be diminished from 75-80% by the Blatchford Score [30] and the Rockall Score to only 11.9%. The rest of the patients would be classified as “moderate” or “urgent” group instead (66.9%). This algorithm will cut down the number of patients requiring immediate endoscopy (< 24 hours) to those who were actually severe. The unnecessary cost of care, both medical personnel, medical instruments, and other medical resources for mild and moderate patients could vastly be reduced.

Our scoring system relied on similar clinical and laboratory parameters, without using endoscopic findings, as the Blatchford Score [21] and the Clinical Rockall Score [5]. It should therefore be usable in many settings where endoscopy is unavailable. Primary or secondary care settings may use this scoring system to selectively transfer “severe” patients to the nearest tertiary care settings. Like other scoring systems developed, our score also need external validation to confirm its discriminative ability and precision.

### Conclusions

Patient profiles might be combined to develop a simple UGIH severity scoring system, which could classify patients into non-urgent, urgent and emergent groups. Small numbers of under- and over-estimations were clinically acceptable. However, its discriminative ability and precision should be validated with a new group of similar patients.
